# Socio-economic factors associated with the 1‑year prevalence of severe pain and pain-related sickness absence in the Austrian population

**DOI:** 10.1007/s00508-017-1222-y

**Published:** 2017-06-21

**Authors:** Ellenor Mittendorfer-Rutz, Thomas Ernst Dorner

**Affiliations:** 1 Department of Clinical Neuroscience, Division of Insurance Medicine, Karolinska Institutet, Berzelius väg 3, 17177 Stockholm, Sweden; 2 Department of Social and Preventive Medicine, Centre for Public Health, Medical University of Vienna, Kinderspitalgasse 15/I, 1090 Vienna, Austria

**Keywords:** Pain prevalence, Socio-economic status, Sickness absence, Chronic mental diseases, Chronic musculoskeletal diseases

## Abstract

**Background:**

The aim of this study was to (1) investigate the relation of socio-economic status (SES), measured as education, occupation, and income, with the 12-month prevalence of severe pain and with pain-related sickness absence, and (2) analyse to what extent sociodemographic and medical factors influence these associations.

**Methods:**

The study population comprised 8084 subjects aged between 15 and 65 years from the Austrian Health Interview Survey in 2006/07. Associations of SES with the 1‑year prevalence of severe pain and sickness absence due to pain in those with severe pain was assessed with logistic regression analysis and adjusted for socio-demographic and chronic medical conditions.

**Results:**

The 1‑year prevalence of severe pain was 33.7%. Among those with severe pain, 32.9% were on sickness absence due to pain. SES was significantly associated with the prevalence of severe pain and even more strongly with sickness absence due to pain. Stepwise adjustment for socio-demographics and medical factors had only marginal effects on these associations. Multivariate odds ratios (ORs) for severe pain were 1.14; 1.18 and 1.32 for low income, blue-collar workers, and low education, respectively. Related ORs for sickness absence due to pain were 1.52; 1.14 and 2.05.

**Conclusions:**

There was an association between SES, particularly measured as educational level, and the prevalence of severe pain, which was even stronger with sickness absence due to pain.

## Introduction

Pain is a public health problem in many European countries [1–3]. It is associated with high societal costs, both, direct and indirect [4, 5]. The leading cause of indirect costs due to pain is related to impaired occupational and vocational functioning [1, 6]. Pain, therefore, ranks among the top diagnostic categories for temporal and permanent loss of productivity [7, 8], like sickness absence [9–11], which often leads to disability pension [1, 9, 12]. In Austria, musculoskeletal disorders, which include many pain-related diagnoses like back pain, the most common pain diagnosis, account for 14% of all sick-leave cases and 22% of all sick-leave days [13].

Socio-economic status (SES), which can be operationalised by education, income, and type of occupation, is one of the major determinants of health [14] and particularly the prevalence of pain [8, 15–17]. Socio-economic differences have been reported to affect sickness absence in general [18], and this is especially pronounced with regard to pain related sickness absence. Previous research on explanatory factors for these SES differences has reported a multitude of factors, including differences in socio-demographics [19]. Additionally, since SES is related to many different chronic somatic non-musculoskeletal diseases, and many chronic diseases are common reasons for sickness absences [13], it is important to take all these relations into account when analysing the association between SES and sickness absence due to pain.

Empirically confirmed models exist on how SES influences health, in particular pain, and sickness absence [20]. Those theories suggest, for instance, that lower SES is on the one hand related to lower general knowledge, health literacy, and material resources, potentially leading to less healthier behaviour, and on the other hand higher work-related physical and mental exposure and increased work demands, leading to poorer work ability [21–24]. Regarding explanatory models within medicine, one can say in the case of sickness absence that “a patient’s work ability is impaired, and that he/she cannot work at 100% capacity” [20]. Still, an employee’s capacity to work in case of reduced work ability is strongly dependent on the type of occupation, which in turn is socio-economically shaped [25, 26]. For example, an assistant nurse is more exposed to heavy manual work than a managing officer. Consequently, in case of severe pain the work ability of the assistant nurse might be more strongly affected and the risk of sick leave higher than might be the case for the managing officer. We therefore hypothesised that the socio-economic gradient would be stronger for sickness absence due to pain than for pain per se. Furthermore, it is not known to date which measures of SES have the strongest relation with pain on the one hand and sickness absence due to pain on the other hand, when controlling for a number of sociodemographic and morbidity factors.

The aim of this study was to:

1. investigate the relation of SES (measured as education, income and type of occupation) with the prevalence of severe pain in the general working-age population,
2. assess the association of SES with pain-related sickness absence in those who had severe pain, and
3. analyse to what extent sociodemographic and medical factors influence these associations.

## Methods

### Study population

The database for this analysis was the Austrian Health Interview Survey (AT-HIS) 2006–2007 [27]. This survey was carried out by Statistics Austria on behalf of the Austrian Federal Ministry of Health, Family and Youth. The survey is a micro-census of a representative sample of the entire Austrian population with the aim to gain knowledge about subjective health, health determinants, and utilisation of the health care system. The questionnaire was designed based on the European Core Health Interview Survey (EC-HIS) [28] and was adapted for Austria by an expert panel. The interviews were conducted face-to-face using computer assisted personal interviewing. The subjects were interviewed between March 2006 and March 2007 by trained interviewers. The sample was stratified by geographic region, with the same number of subjects being included from each region. In order to account for the stratification of the sample, the data were weighted by geographic region, age, and sex. Missing values were systematised.

The gross sample size was 25,130 subjects aged over 15 years. Of these, 9656 subjects were excluded (5709 subjects refused or discontinued the interview, 3308 were excluded due to difficulties in contacting them or because of deficiency in the German language, and for 639 cases data quality was insufficient). The information of a total of 15,474 subjects was eligible for the analysis, thus the response rate was 63.1%. For this analysis, only data for subjects aged 15–64 years, who were gainfully employed at the time of questioning, were used. Therefore, the study population comprised 8084 subjects.

### Outcome measures

Outcome measures comprised 12-month prevalence of severe pain and 12-month prevalence of sickness absence due to severe pain. These were assessed with the following questions: “Did you suffer from severe pain in one or more than one body site during the last 12 months?” and “Did you have sickness absence due to this pain in the last 12 months?”

### Exposure and covariates

Education, type of occupation, and income were used as socio-economic variables. Level of education was measured with three levels: primary education (up to the age of 15 years), secondary education (apprenticeship, vocational school or secondary school with the Austrian school leaving exam “Matura”), and tertiary education (university or any other vocational training after the “Matura”). Occupation was assessed in three categories: self-employed (which also included freelancers and farmers), white-collar workers (which also comprised civil servants), and blue-collar workers. Income was indexed in three groups of net household income per month, divided by the number of household residents. Categories were built based on tertiles: 1–600 €, 601–1500 €, and 1501 € and more. The socio-demographic variables age, sex, and family status were also included in the analysis. Age was assessed in three categories: 15–34 years, 35–49 years, and 50–64 years. Family status was dichotomized as having (married or in partnership) or not having (single, divorced, widowed) a partner.

In the AT-HIS, 12-month prevalence for medical factors, i. e. several diseases and health complaints, was investigated. Information on somatic non-musculoskeletal disease comprised the following 14 diseases:

1. allergic bronchial asthma,
2. other forms from asthma,
3. other allergies,
4. diabetes mellitus,
5. cataract,
6. tinnitus,
7. hypertension,
8. myocardial infarction,
9. stroke,
10. chronic bronchitis or emphysema,
11. urinary incontinence,
12. gastric or duodenal ulcer,
13. cancer, and
14. migraine.

For the variable chronic musculoskeletal disease, the following diagnostic groups were included:

1. osteoporosis,
2. osteoarthritis, and
3. chronic spinal disorders.

Finally, mental disease was defined as anxiety and/or depression. All individuals with chronic conditions were asked whether or not they had occurred in the last 12 months. The variables somatic non-musculoskeletal disease and musculoskeletal disease were dichotomised in two variables with either having at least one of the respective diseases or none of them.

### Statistical analyses

Bivariate analyses were undertaken by means of cross-tabs, and group differences were assessed with the Z‑test. Binary logistic regression models were applied. Severe pain in all subjects, as well as having been on sickness absence due to pain in the sub-sample with severe pain were used as dependent variables. The different measures of SES were introduced as categorical independent variables. In model I, crude odds ratios (OR) and 95% confidence intervals (95% CI) were computed. In model II, we adjusted for age, sex, and family status. In model III, additional adjustments were due to mental diseases, in model IV, we additionally adjusted for somatic non-musculoskeletal diseases, and in model V, chronic musculoskeletal diseases were additionally adjusted for. The results of all logistic regression models are presented as odds ratios (OR) and 95% confidence intervals (95% CI). Moreover, R^2^ were calculated at each adjustment step in order to evaluate the extent of the model fit. For statistical analyses, IBM SPSS Statistics 21 was used.

The secondary analysis of the AT-HIS database that was used for this study was approved by the Ethics Committee of the Medical University Vienna (EC # 770/2011).

## Results

As shown in Table 1, one third of the participants was affected by severe pain within the last 12 months. Of these, one third was on sickness absence due to pain during the same time span. Subjects with higher income had lower rates of severe pain, and with increasing income the proportion of those on sickness absence due to pain decreased gradually. Occupation was mildly associated with the 12-month prevalence of pain and clearly associated with the proportion of individuals on sickness absence due to pain. Similarly, education was gradually and inversely associated with the proportion of those suffering from severe pain and the proportion of those being on sickness absence.

**Table 1:** One-year prevalence of severe pain and sickness absence due to pain in those with severe pain by different socio-demographic, socio-economic and medical factors

	*N*	Percentage of subjects with severe pain within the last 12 months	Percentage of subjects with severe pain on sickness absence due to pain in last 12 months
Total	8084	33.7	32.9
*Income*			
0–1500	2298	33.6^a,b^	38.0^a^
1501–3000	3807	34.9^b^	32.6^b^
3001+	1979	31.3^a^	27.5^c^
*Occupation*			
Blue-collar workers	2340	36.9^a^	38.8^c^
Self-employed	999	34.8^a,b^	16.9^a^
White-collar workers	4745	31.8^b^	33.3^b^
*Education*			
Primary education	1235	37.6^a^	39.9^a^
Secondary education	5804	33.4^b^	32.9^b^
Tertiary education	1045	30.7^b^	23.1^c^
*Sex*			
Male	4579	31.3^a^	35.8^a^
Female	3505	36.8^b^	29.8^b^
*Age*			
15–34	2813	25.4^a^	35.6^a^
35–49	3721	35.5^b^	32.6^a^
50–64	1550	44.3^c^	30.9^a^
*Family status*			
Single, divorced, widowed	2319	28.8^a^	36.9^a^
Married or in partnership	5764	35.6^b^	31.7^b^
*Chronic somatic non-musculoskeletal disorders*			
Yes	3122	47.5^a^	33.4^a^
No	4963	25.0^b^	32.4^b^
*Chronic musculoskeletal disorders*			
Yes	2556	65.0^a^	34.7^a^
No	5527	19.2^b^	30.3^b^
*Chronic mental disorders*			
Anxiety/depression	296	64.2^a^	42.1^a^
No anxiety/depression	7789	35.8^b^	32.3^b^

^a, b, c^The letters following the percentages represent a subset of the variable category that is significantly different at a significance level of *p* < 0.05 if it is not the same letter

Male sex was associated with a lower proportion of subjects with severe pain, but if pain occurred, men showed a higher proportion of sickness absence. Higher age was associated with a higher chance of severe pain, but not with being on sickness absence. All three parameters of chronic diseases (somatic non-musculoskeletal, musculoskeletal, and mental diseases) were associated with a higher chance for severe pain, and a higher proportion of subjects being on sickness absence (Table 1).

All three socio-economic factors were significantly associated with the risk of suffering from severe pain in the logistic regression analysis (Table 2). In the crude model, ORs ranged from 1.11 to 1.36. The estimates for R^2^, however, were very low: 0.1%; 0.2%, and 0.3% for income, education, and type of occupation, respectively. Stepwise adjustment for mental, somatic non-musculoskeletal, and particularly chronic musculoskeletal disorders lowered the estimates for ORs. The introduction of these factors was associated with considerable increases in R^2^ estimates. In the final model, income was no longer significantly related to severe pain. ORs for severe pain in blue-collar workers and those with low education were 1.18, and 1.32, respectively.

**Table 2:** Odds ratios (OR) and 95% confidence intervals (95% CI) from binary logistic regression analysis with severe pain in the last 12 months (yes vs. no) as a dependent variable, and three measures of socio-economic status (SES) as independent variables, stepwise adjusted for socio-demographic and medical factors

	Model I			Model II			Model III			Model IV			Model V		
	OR	95% CI	R^2^	OR	95% CI	R^2^	OR	95% CI	R^2^	OR	95% CI	R^2^	OR	95% CI	R^2^
**Income**															
0–1500	1.11	0.98–1.26	0.001	1.23	1.08–1.40	0.040	1.19	1.04–1.37	0.056	1.22	1.06–1.40	0.105	1.14	0.98–1.32	0.283
1501–3000	1.18	1.05–1.32		1.22	1.09–1.38		1.20	1.07–1.36		1.22	1.08–1.37		1.11	0.97–1.27	
3001+	1	–		1	–		1	–		1	–		1	–	
*Sex*															
Female	–	–	0.001	1.28	1.17–1.41	0.040	1.24	1.13–1.37	0.056	1.13	1.02–1.24	0.105	1.13	1.02–1.26	0.283
Male	–	–		1	–		1	–		1	–		1	–	
*Age (5 years)*	–	–		1.16	1.13–1.18		1.15	1.12–1.18		1.13	1.11–1.16		1.05	1.02–1.08	
*Family situation*															
Having a partner	–	–	0.001	1.14	1.01–1.27	0.040	1.16	1.04–1.30	0.056	1.14	1.02–1.24	0.105	1.09	0.96–1.24	0.283
No partner	–	–		1	–		1	–		1	–		1	–	
*CMD* ^*a*^	–	–		–	–		3.35	2.62–4.29		2.77	2.16–3.57		2.07	1.57–2.73	
*CSNMSD* ^*b*^	–	–		–	–		–	–		2.38	2.16–2.62		1.93	1.73–2.15	
*CMSD* ^*c*^	–	–		–	–		–	–		–	–		6.53	5.85–7.29	
**Occupation**															
Blue-collar workers	1.25	1.13–1.39	0.003	1.45	1.30–1.61	0.045	1.40	1.26–1.56	0.060	1.44	1.28–1.61	0.110	1.18	1.04–1.33	0.283
Self-employed	1.15	0.99–1.32		1.04	0.90–1.21		1.05	0.90–1.22		1.07	0.92–1.24		1.04	0.88–1.22	
White-collar workers	1	–		1	–		1	–		1	–		1	–	
*Sex*															
Female	–	–	0.003	1.37	1.25–1.51	0.045	1.32	1.20–1.46	0.060	1.20	1.09–1.33	0.110	1.17	1.05–1.30	0.283
Male	–	–		1	–		1	–		1	–		1	–	
*Age (5 years)*	–	–		1.16	1.14–1.19		1.15	1.13–1.18		1.14	1.11–1.17		1.05	1.03–1.08	
*Family situation*															
Having a partner	–	–	0.003	1.13	1.01–1.26	0.045	1.15	1.03–1.29	0.060	1.13	1.01–1.27	0.110	1.08	0.95–1.22	0.283
No partner	–	–		1	–		1	–		1	–		1	–	
*CMD* ^*a*^	–	–		–	–		3.24	2.53–4.16		2.68	2.08–3.49		2.04	1.55–2.70	
*CSNMSD* ^*b*^	–	–		–	–		–	–		2.39	2.17–2.63		1.94	1.74–2.15	
*CMSD* ^*c*^	–	–		–	–		–	–		–	–		6.46	5.79–7.21	
**Education**															
Primary education	1.36	1.14–1.62	0.002	1.59	1.33–1.90	0.042	1.56	1.30–1.87	0.058	1.67	1.35–1.96	0.108	1.32	1.08–1.61	0.283
Secondary education	1.13	0.98–1.30		1.25	1.08–1.44		1.26	1.09–1.46		1.34	1.14–1.54		1.24	1.05–1.45	
Tertiary education	1	–		1	–		1	–		1	–		1	–	
*Sex*															
Female	–	–	0.002	1.28	1.17–1.41	0.042	1.24	1.13–1.37	0.058	1.13	1.02–1.24	0.108	1.14	1.02–1.28	0.283
Male	–	–		1	–		1	–		1	–		1	–	
*Age (5 years)*	–	–		1.16	1.13–1.19		1.15	1.13–1.18		1.14	1.11–1.17		1.05	1.03–1.08	
*Family situation*															
Having a partner	–	–	0.002	1.12	1.00–1.25	0.042	1.15	1.02–1.28	0.058	1.13	1.00–1.26	0.108	1.08	0.96–1.22	0.283
No partner	–	–		1	–		1	–		1	–		1	–	
*CMD* ^*a*^	–	–		–	–		3.36	2.67–4.30		2.79	2.16–3.59		2.09	1.59–2.76	
*CSNMSD* ^*b*^	–	–		–	–		–	–		2.39	2.17–2.63		1.94	1.74–2.16	
*CMSD* ^*c*^	–	–		–	–		–	–		–	–		6.50	5.83–7.25	

*Model I* crude analysis

*Model II* adjusted for sex, age, and family status

*Model III* like model II and additionally adjusted for chronic mental diseases

*Model IV* like model III and additionally adjusted for chronic somatic non-musk diseases

*Model V* like model IV and additionally adjusted for chronic musculoskeletal diseases

^a^Chronic mental disease

^b^Chronic somatic non-musculoskeletal disease

^c^Chronic musculoskeletal disease

According to logistic regression analyses in Table 3, the socio-economic variables were clearly associated with pain related sickness absence in those with severe pain. ORs ranged from 1.27 to 2.22 and R^2^ estimates from 0.9% to 3%. Of note, being self-employed was associated with a lower risk of sickness absence due to pain. There was almost no effect on the estimates for the ORs and only a small effect on the estimates for the R^2^ when stepwise adjusted for socio-demographic factors and diseases. In the final model, ORs for low income and low education with respect to sickness absence due to pain were 1.52 and 2.05, respectively.

**Table 3:** Odds ratios (OR) and 95% confidence intervals (95% CI) from binary logistic regression analysis with regard to being on sickness absence due to pain in the last 12 months as a dependent variable (yes vs. no), and three measures of socio-economic status as independent variables in those with severe pain, stepwise adjusted for socio-demographic and medical factors

	Model I			Model II			Model III			Model IV			Model V		
	OR	95% CI	R^2^	OR	95% CI	R^2^	OR	95% CI	R^2^	OR	95% CI	R^2^	OR	95% CI	R^2^
**Income**															
0–1500	1.62	1.29–2.03	0.009	1.57	1.24–1.98	0.018	1.54	1.22–1.95	0.022	1.54	1.22–1.95	0.023	1.52	1.20–1.92	0.026
1501–3000	1.27	1.03–1.57		1.26	1.02–1.55		1.24	1.01–1.54		1.24	1.01–1.54		1.22	0.99–1.51	
3001+	1	–		1	–		1	–		1	–		1	–	
*Sex*															
Female	–	–	0.009	0.74	0.63–0.87	0.018	0.72	0.61–0.85	0.022	0.71	0.60–0.84	0.023	0.71	0.60–0.84	0.026
Male	–	–		1	–		1	–		1	–		1	–	
*Age (5 years)*	–	–		0.97	0.93–1.01		0.97	0.93–1.00		0.97	0.93–1.00		0.96	0.92–0.99	
*Family situation*															
Having a partner	–	–	0.009	0.86	0.71–1.04	0.018	0.87	0.72–1.06	0.022	0.87	0.72–1.06	0.023	0.86	0.70–1.04	0.026
No partner	–	–		1	–		1	–		1	–		1	–	
*CMD* ^*a*^	–	–		–	–		1.57	1.16–2.13		1.56	1.14–2.12		1.51	1.11–2.06	
*CSNMSD* ^*b*^	–	–		–	–		–	–		1.05	0.89–1.24		1.04	0.88–1.23	
*CMSD* ^*c*^	–	–		–	–		–	–		–	–		1.27	1.06–1.50	
**Occupation**															
Blue-collar workers	1.27	1.07–1.51	0.030	1.20	1.00–1.43	0.037	1.18	0.98–1.41	0.041	1.18	0.98–1.41	0.041	1.14	0.95–1.37	0.044
Self-employed	0.40	0.30–0.55		0.41	0.30–0.55		0.41	0.30–0.55		0.41	0.30–0.55		0.41	0.30–0.55	
White-collar workers	1	–		1	–		1	–		1	–		1	–	
*Sex*															
Female	–	–	0.030	0.77	0.65–0.91	0.037	0.75	0.63–0.88	0.041	0.74	0.63–0.88	0.041	0.74	0.62–0.88	0.044
Male	–	–		1	0.95–1.02		1	–		1	–		1	–	
*Age (5 years)*	–	–		0.98	–		0.98	0.94–1.02		0.98	0.94–1.02		0.97	0.93–1.01	
*Family situation*															
Having a partner	–	–	0.030	0.82	0.68–1.00	0.037	0.84	0.69–1.01	0.041	0.84	0.69–1.01	0.041	0.82	0.68–1.00	0.044
No partner	–	–		1	–		1	–		1	–		1	–	
*CMD* ^*a*^	–	–		–	–		1.52	1.12–2.07		1.51	1.11–2.07		1.47	1.08–2.01	
*CSNMSD* ^*b*^	–	–		–	–		–	–		1.03	0.87–1.22		1.02	0.86–1.21	
*CMSD* ^*c*^	–	–		–	–		–	–		–	–		1.26	1.06–1.50	
**Education**															
Primary education	2.22	1.61–3.05	0.013	2.16	1.58–2.98	0.023	2.13	1.54–2.94	0.027	2.13	1.54–2.94	0.027	2.05	1.48–2.83	0.030
Secondary education	1.64	1.24–2.16		1.56	1.18–2.06		1.56	1.18–2.07		1.57	1.19–2.07		1.53	1.16–2.03	
Tertiary education	1	–		1	–		1	–		1	–		1	–	
*Sex*															
Female	–	–	0.013	0.74	0.63–0.87	0.023	0.72	0.61–0.85	0.027	0.72	0.61–0.85	0.027	0.72	0.61–0.85	0.030
Male	–	–		1	–		1	–		1	–		1	–	
*Age (5 years)*	–	–		0.98	0.94–1.01		0.97	0.94–1.01		0.97	0.93–1.01		0.96	0.92–1.00	
*Family situation*															
Having a partner	–	–	0.013	0.81	0.67–0.98	0.023	0.82	0.68–1.00	0.027	0.82	0.68–1.00	0.027	0.81	0.67–0.98	0.030
No partner	–	–		–	–		1	–		1	–		1	–	
*CMD* ^*a*^	–	–		–	–		1.58	1.16–2.14		1.56	1.15–2.13		1.52	1.12–2.08	
*CSNMSD* ^*b*^	–	–		–	–		–	–		1.04	0.88–1.23		1.03	0.87–1.22	
*CMSD* ^*c*^	–	–		–	–		–	–		–	–		1.23	1.04–1.47	

*Model I* crude analysis

*Model II* adjusted for sex, age, and family status

*Model III* like model II and additionally adjusted for chronic mental diseases

*Model IV* like model III and additionally adjusted for chronic somatic non-musculoskeletal diseases

*Model V* like model IV and additionally adjusted for chronic musculoskeletal diseases

^a^Chronic mental disease

^b^Chronic somatic non-musculoskeletal disease

^c^Chronic musculoskeletal disease

## Discussion

In our analyses we found that socio-economic adversity was more strongly related to sickness absence due to pain in individuals with severe pain than to severe pain itself. Adjustment for socio-demographic and medical factors only marginally affected these associations for both outcome measures. The highest ORs were found for low education, but occupation was most strongly associated with both pain and sickness absence due to pain.

Associations of socio-economic factors with both severe pain and sickness absence due to pain were found as previously reported [8, 15–19]. We could now also show that there was a stronger socio-economic gradient with regard to sick leave due to pain than with severe pain per se. Moreover, socio-demographic and morbidity factors explained much less of the variability of sickness absence due to pain than in the prevalence of severe pain itself. These findings suggest that there is an even stronger socio-economic gradient in pathways to reduced work capacity due to pain than in pathways to severe pain. We could further show that other socio-demographic factors and chronic diseases only marginally affected these associations. Factors which can additionally influence the decision to take sickness absence due to severe pain include work-related factors and health behaviour, which are in turn socio-economically determined. Individuals with lower socio-economic status might be more often exposed to poor psychosocial and physical working conditions [21, 22]. They might also have fewer possibilities to adapt their working situation in order to limit the risk of work incapacity. Moreover, adverse health behaviour, including infrequent physical exercise and high alcohol use, has been shown to be associated with sickness absence and socio-economic adversities [23, 24].

As in previous studies [15, 29, 30], we found a stronger relationship to education and occupation with pain and sickness absence due to pain rather than to income. Similarly, education was found to be more related to health behaviour than other measures of SES in the Austrian population [14]. But, of course, those three factors of SES are often interrelated [29]. Different reasons for the high impact of education on sickness absence have been discussed in the literature [29]. Those reasons include the fact that higher education gives people more understanding, health literature, and health knowledge and helps patients to cope better with the disease. Another possible explanation is that better health is not the consequence, but rather the cause of higher education and selection could provide healthier people with better opportunities for education. Additionally, education determines occupational position and income to a large extent and those factors might lead to differences in sickness absence [29].

There are several plausible reasons why occupation is strongly related to sickness absence. Self-employed subjects often do not strive to be granted sickness absence because they fear loss of income. This explains why self-employed people were the occupation group in our analysis with the lowest rate of sickness absence in subjects with severe pain. However, we also found that blue-collar workers with severe pain were granted sickness absence more often than white-collar workers. This can be due to the fact that blue-collar workers are more frequently exposed to heavy manual work and, if severe pain occurs, it has a higher influence on work capacity in those subjects. But also psychosocial working conditions, which affect health status and modify work ability, differ between occupational groups and can contribute to differences in sickness absence [29].

We found a remarkable sex difference for being on sickness absence due to pain in those suffering from severe pain. Men had a higher prevalence than did women. However, most studies found higher sickness absence rates in women than in men [31]. In contrast to our analysis, the overall sickness absence rate was examined in those studies and not the sickness absence rate in those with a given medical condition. Different working conditions for men and women might contribute to the sex difference. Men might work more often in upper white-collar or managerial positions, or are self-employed and might more often have a higher income, whereas women might work more often in lower white-collar positions and have a lower income [31]. Therefore, work ability in different professions might be differently affected by severe pain, which could explain the differential risk for sickness absence.

This study has several strengths, including a large and representative sample of the Austrian population with a considerable number of measured characteristics. Moreover, unlike most surveys, detailed data on pain was available. We used three different measures for the operationalisation of SES. A number of limitations should also be mentioned. Underreporting is likely in the measures of chronic diseases. Therefore, the possibility of residual confounding in the multivariate models adjusting for chronic diseases cannot be excluded. The interpretation of cross-sectional studies is hampered, since causal associations cannot be drawn. The validity of self-reported data on sickness absence has been investigated in several previous studies with divergent findings [32, 33]. The possibility of recall bias needs to be mentioned as a further limitation.

In conclusion, in our analysis we found a clear socio-economic gradient in the prevalence of severe pain, but an even stronger socio-economic gradient for being on sick leave due to pain in those with severe pain independent of the effect of socio-demographics and morbidity. Education and occupations seem to be more strongly related to both outcome measures than income.
